# Cytokine production and signalling in human THP-1 macrophages is dependent on *Toxocara canis* glycans

**DOI:** 10.1007/s00436-019-06405-8

**Published:** 2019-08-08

**Authors:** Ewa Długosz, Katarzyna Basałaj, Anna Zawistowska-Deniziak

**Affiliations:** 1 Division of Parasitology, Department of Preclinical Sciences, Faculty of Veterinary Medicine, Warsaw University of Life Sciences-SGGW, Ciszewskiego 8, 02-786 Warsaw, Poland; 2 W. Stefański Institute of Parasitology, Twarda 51/55, 00-818 Warsaw, Poland

**Keywords:** *Toxocara*, Antigens, Macrophages, Cytokines, Kinases, Signalling

## Abstract

The effect of *Toxocara canis* antigens on cytokine production by human THP-1 macrophages was studied in vitro. *Toxocara* Excretory–Secretory products (TES) and recombinant mucins (Tc-MUC-2, Tc-MUC-3, Tc-MUC-4, and Tc-MUC-5) as well as deglycosylated forms of these antigens were used in the study. TES products stimulated macrophages to produce the innate proinflammatory IL-1β, IL-6, and TNF-α cytokines regardless of the presence of glycans. Recombinant mucins induced glycan-dependent cytokine production. Sugar moieties led to at least 3-fold higher production of regulatory IL-10 as well as proinflammatory cytokines. The presence of glycans on mucins also affected the downstream signalling pathways in stimulated cells. The most prominent difference was noted in AKT and AMPK kinase activation. AKT phosphorylation was observed in cells stimulated with glycosylated mucins, whereas treatment with deglycosylated antigens led to AMPK phosphorylation. MAP kinase family members such as JNK and p38 and c-Jun transcription factor were phosphorylated in both cases what suggests that toll-like receptor signalling may be involved in mucin-treated macrophages. This pathway is however modified by other signalling molecules as only mucins containing intact sugars significantly induced the production of cytokines.

## Introduction

*Toxocara canis* and *Toxocara cati*, the dog and the cat roundworm respectively, are parasitic nematodes with great zoonotic potential. Toxocariasis is prevalent across the globe, with children especially at risk of infection. The incidence of *T. canis* serum-positive individuals varies from 1.6% in Japan and 2.4% in Denmark, up to 63% in Bali, 86% in Saint-Lucia, and almost 93% in La Reunion (Magnaval et al. 2001; Ma et al. 2018).

Upon infection of the human host, *Toxocara* larvae migrate through various tissues leading to different clinical syndromes: visceral larva migrans (VLM), ocular larva migrans (OLM), and cerebral or neurotoxocariasis (NT), which is considered as the most cryptic of all disease syndromes (Holland 2017). However, the most common syndrome and the most difficult to diagnose is covert toxocariasis (CT) with many unspecific symptoms such as arthralgia, lymphadenopathy, fever, or headaches. *Toxocara* worms also contribute to the development of allergic diseases, including asthma, chronic urticaria, or angioedema (Magnaval et al. 2001; Pinelli et al. 2008).

Many studies concerning the interaction between *Toxocara* and the immune system come from experiments conducted in murine model of the disease (Kuroda et al. 2001; Pinelli et al. 2008; Faz-López et al. 2013; Długosz et al. 2015). These reports show that a Th2 response is predominant. T cells produce IL-4 and IL-5 cytokines after contact with *T. canis* excretory-secretory antigens (TES). At the same time, IFN-γ and TNF-α are downmodulated and regulatory cytokines IL-10 and TGF-β are upregulated (Kuroda et al. 2001; Fan et al. 2004). Th1 and Th17 immune responses are strongly inhibited in mice infected with *T. canis* comparing with control animals (Długosz et al. 2015). This strong Th2 response allows for the establishment of heavy parasite loads in different tissues, but at the same time, it protects mice from the development of severe pathology in lungs (Faz-López et al. 2013).

Previous reports show that healthy human T cells produce significant amounts of IL-4 and IL-5, but no IL-2 or IFN-γ after stimulation with TES (Del Prete et al. 1991). Inuo et al. (1995) reported that human PBMC proliferate in response to *Toxocara* adult worm antigen (TcAg) and that their differentiation into Th2 cells is not obvious as IL-2 and IFN-γ mRNA expression was noted along with the expression of IL-4 and IL-5 mRNA. Moreover TcAg was also shown to stimulate the proliferation of human B cells (Wang et al. 1995). In a more recent clinical study, the level of cytokines in sera of patients with VLM was analysed. The study shows that in children with hepatic involvement, IL-4, IL-6, and IL-10 levels are elevated together with eosinophilia and IgE hyperimmunoglobulinemia (Mazur-Melewska et al. 2014). The authors suppose that the pathology of VLM results from the imbalance between pro-inflammatory and anti-inflammatory cytokines in these patients.

Human toxocariasis is considered as a neglected parasitic infection. More efforts should be made to enrich our understanding of the infection process in the human host. Using the feasible in vitro models and comparing the results with those obtained in the mouse model will bring us forward to fill many gaps in our knowledge of toxocariasis. Therefore, the aim of the study was the analysis of human macrophage in vitro cytokine response to *T*. *canis* antigens and the evaluation of the possible signalling pathways involved in this response.

## Materials and methods

### Antigen preparation

Collection of TES from in vitro larvae culture and the production of recombinant *T. canis* mucins in *Pichia pastoris* yeast is described elsewhere (Długosz et al. 2015). In order to eliminate sugar moieties, TES and recombinant mucins were treated with sodium metaperiodate according to the method described by Tawill et al. (Tawill et al. 2004) and the deglycosylation process was monitored by protein electrophoresis (Długosz and Wisniewski 2016).

### Cell culture

The THP-1 human monocyte cell line was purchased from the American Type Culture Collection. Cells were maintained in culture medium (RPMI 1640 supplemented with 10% foetal bovine serum, 2-mM glutamine, 100-U/ml penicillin, 100-μg/ml streptomycin) at 37 °C in a humidified atmosphere of 5% CO_2_. The cells were seeded into 24-well plates at a concentration of 1 × 10^6^/ml in a volume of 1 ml/well. The cells were differentiated into macrophages by the addition of 100-ng/ml phorbol 12-myristate 13-acetate (PMA) for 72 h. After differentiation, the cells were washed twice with fresh media w/o PMA and stimulated with ES (5 μg/ml), a mixture of recombinant mucins (1.25 μg/ml of each Tc-MUC-2, Tc-MUC-3, Tc-MUC-4 and Tc-MUC-5) or individual mucins (1.25 μg/ml) with or without lipopolysaccharide (LPS) (100 ng/ml). The same stimulation procedures were performed with deglycosylated antigens. The stimulation culture media were collected after 24 h and stored at − 70 °C until use.

### Cytokine assays

The concentrations of TNF-α, IL-1β, IL-6, IL-12p70, and IL-10 were determined using the commercial ELISA kits OptEIA ™ Set Human (BD Biosciences) and IL-23 using DuoSet ELISA (R&D Systems) according to manufacturer instruction. Experiments were performed independently in triplicate. Statistical analysis was performed by Student’s *t* test. A value of *p* < 0.05 was considered to be significant. Analysis was done using Statgraphics Plus 4.1 software. Results are shown as mean ± SD.

### Phospho-kinase array

The phospho-antibody array analysis was performed using the Proteome Profiler Human Phospho-Kinase Array Kit from R&D Systems according to the manufacturer’s instructions. Proteins were isolated from THP-1 macrophages stimulated for 24 h with a mixture of recombinant glycosylated and deglycosylated mucins (1.25 μg/ml of each Tc-MUC-2, Tc-MUC-3, Tc-MUC-4, and Tc-MUC-5) and from unstimulated control cells. The protein concentration was measured using a Pierce ™ BCA Protein Assay Kit (Thermo Scientific). Pre-blocked nitrocellulose membranes of the Human Phospho-Kinase Arrays were incubated with equal amounts of cellular extract overnight at 4 °C on a rocking platform. The membranes were washed three times with 1× Wash Buffer (R&D Systems) to remove the unbound proteins and were then incubated with a mixture of biotinylated detection antibodies and streptavidin-HRP antibodies. Chemiluminescent detection reagents were applied to detect spot densities. Membranes were exposed to X-ray film for 3, 5, and 10 min. Array images were analysed using image analysis software Quantity One (Biorad).

## Results and discussion

The interaction between *Toxocara* parasites and the immune system of the human host is not fully understood. Therefore we decided to analyse the effect of roundworm antigens on cytokine release by human macrophages since these cells are reliable model for other organisms (Alvarado et al. 2017; Bąska et al. 2017). Macrophages are a heterogeneous group of immune cells and their phenotype and functions are regulated by the surrounding micro-environment (reviewed in Shapouri-Moghaddam et al. 2018). We used the THP-1 human leukaemia monocytic cell line as it has been used with success in studies concerning monocyte/macrophage functions, mechanisms, and signalling pathways during other helminth infections (Johnston et al. 2010; Bąska et al. 2013; Silva-Álvarez et al. 2016; Zawistowska-Deniziak et al. 2017).

**Fig. 1:**
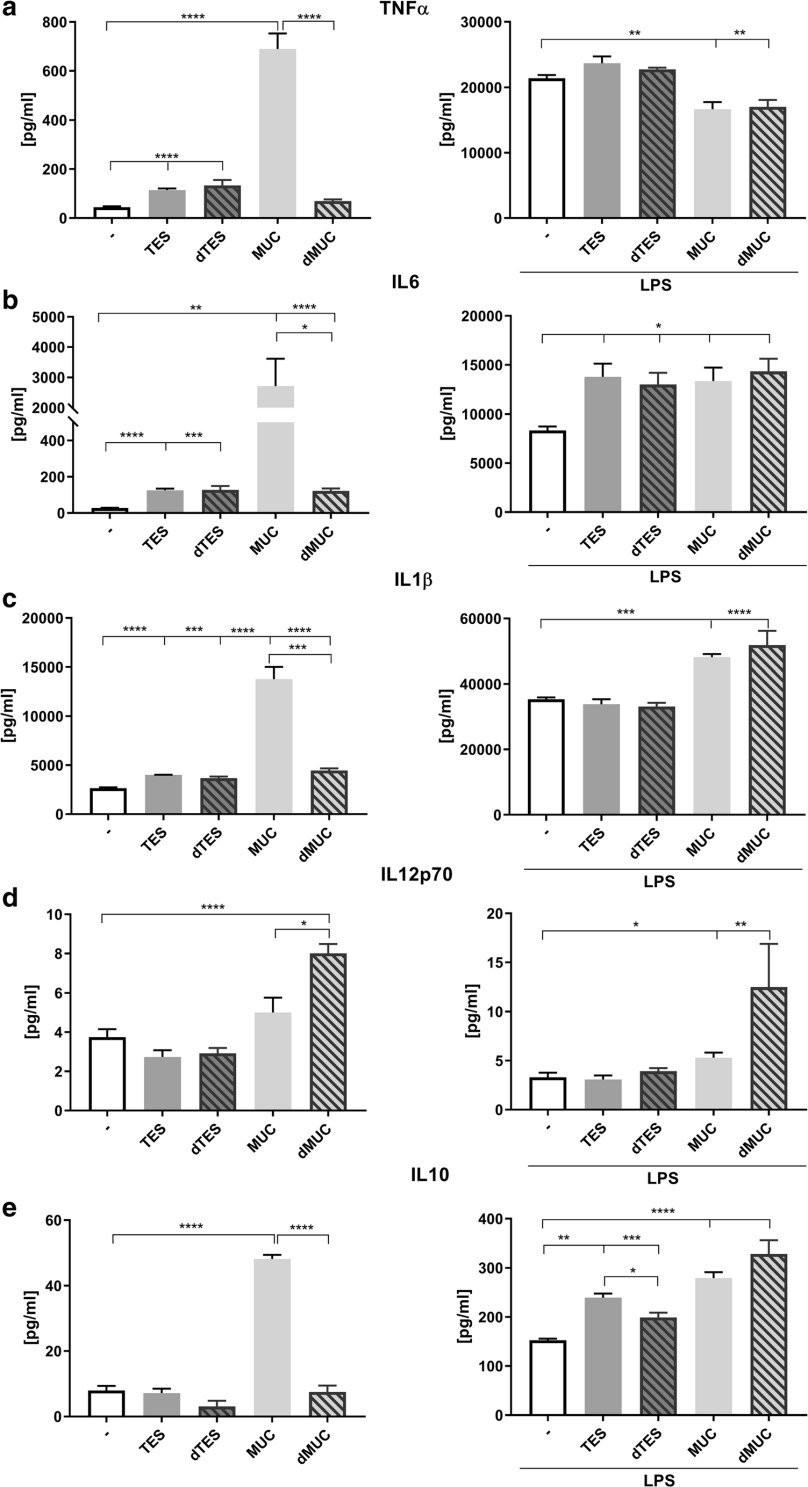
Cytokine production by THP-1 macrophages stimulated with: TES; deglycosylated TES (dTES); recombinant mucins (MUC); deglycosylated mucins (dMUC), and unstimulated control cells with and without simultaneous LPS treatment. Results are presented as mean of calculated concentration [pg/ml] ± SD. Statistical analysis was performed by Student’s *t* test. A value of *P* < 0.05 was considered to be significant. Significant differences between groups are marked with: *p < 0.05, **p < 0.01, ***p < 0.001, **** *p* < 0.0001, **a**-**e**

Stimulation of THP-1 cells with *T. canis* molecules, especially mucins resulted in the production of TNF-α, IL-1β, IL-6, IL-10, and very low amounts of IL-12p70 (Fig. 1). IL-23 was detected only after additional LPS stimulation (data not shown), and no significant differences between groups were noted.

Many helminth parasites induce IL-10 production and different sources of this immunoregulatory cytokine have been described. For example, IL-10 producing regulatory T cells are induced by *Schistosoma*-modulated dendritic cells (Van der Kleij et al. 2002) whilst filarial ES-62 antigen induces IL-10 production by peritoneal B1 lymphocytes (Wilson et al. 2003). Macrophages showing a regulatory phenotype after induction by helminth cystatins are another source of IL-10 (Klotz et al. 2011). *T. canis* ES antigens stimulate the production of IL-10 by splenocytes and macrophages of infected, but not control mice (Kuroda et al. 2001; Długosz et al. 2015). In this study, the amount of IL-10 secreted by THP-1 cells after stimulation with TES did not differ from the control; however, it was significantly higher after stimulation with recombinant mucins (Fig. 1). All investigated molecules increased IL-10 production by macrophages when costimulated with LPS.

We noted a significant increase in IL-6 production after stimulation with all tested antigens, and surprisingly the amount secreted by cells treated with mucins was about 25-fold higher (Fig. 1). Serum IL-6 level in children with VLM was also significantly higher comparing with healthy controls (Mazur-Melewska et al. 2014). IL-6 production by *T. canis*-infected mouse splenocytes was also highly upregulated after TES and mucin stimulation; however, in this case, mucins were less effective (Długosz et al. 2015). On contrary, Kuroda et al. (2001) did not detect the production of IL-6 by murine macrophages.

The role of IL-6 in *T. canis* infections remains to be determined. It is not likely that it would favour parasite survival by restricting the Th2 response as it was described in the case of *Heligmosomoides polygyrus* in mice (Smith and Maizels 2014). During *Toxocara* infection, the limitation of Th2 response is not beneficial to either the parasite, as worm number is reduced, nor for the host as the pathology is augmented (Faz-López et al. 2013). IL-6 is a cytokine with a wide range of functions, one of which is the regulation of acute and chronic inflammation (Naka et al. 2002). Together with TGF-β, it also plays a pivotal role in favouring Th17 differentiation (Basso et al. 2009). Studies from the mouse model of toxocariasis concerning the involvement of Th17 response are not convergent. IL-17 was noted in mouse sera at 14-day post-infection (Resende et al. 2015), on contrary, it was not produced by splenocytes at 21-day post-infection (Długosz et al. 2015). There are no reports on IL-17 production during the course of infection in humans; therefore, the development of Th17 cells and the possible role of IL-6 during human toxocariasis remains to be investigated.

TNF-α was also upregulated in THP-1 cells, after treatment with *T. canis* molecules (Fig. 1). Previously secretion of TNF-α by mouse immune cells was shown to be downmodulated (Kuroda et al. 2001; Długosz et al. 2015). Here we show that even though mucins alone increase TNF-α production, they have the opposite effect after simultaneous cell stimulation with LPS.

A similar pattern of cytokine production was observed for IL-1β (Fig. 1), with the exception that cell stimulation with mucins further upregulated its secretion after LPS treatment. Production of IL-1β by epithelial cells was noted during *Trichinella spiralis* (Li et al. 1998) and *H. polygyrus* infections (Zaiss et al. 2013). Increased production of IL-1β suppresses the release of innate cytokines, resulting in suboptimal type 2 immunity and allowing pathogen chronicity (Zaiss et al. 2013).

Macrophages polarise into two major populations with different functions. Classically activated or inflammatory macrophages (M1) are typically induced by Th1 cytokines, such as IFN-γ and TNF-α or by bacterial LPS. Alternatively activated or anti-inflammatory (M2) macrophages are polarised by Th2 cytokines IL-4 and IL-13. M2 macrophages were further divided into four different subsets (M2a, M2b, M2c, and M2d) depending on the activating stimulus (Martinez et al. 2008). During our study THP-1 macrophages secreted both pro-inflammatory (IL-1β, IL-6, and TNF-α) and regulatory (IL-10) cytokines. Such cytokine profile is typical for so-called M2b macrophages. These cells are induced by immune complexes and TLR ligands and take part in Th2 activation and immunoregulation (Shapouri-Moghaddam et al. 2018).

M2 macrophages are induced by many parasite antigens (Hewitson et al. 2009; Peon et al. 2013), and their presence was also confirmed in *Toxocara* infected tissues (Faz-López et al. 2013). M2b macrophages were so far described only in case of *Leishmania major* (Filardy et al. 2018) and *Acanthocheilonema viteae* (Klotz et al. 2011) infections.

Our studies show that *T. canis* mucins stimulate THP-1 cells to produce significantly larger amounts of cytokines compared with unstimulated cells. Mucins are group of heavily O-glycosylated high molecular weight proteins constituting the TES-120 fraction (Gems and Maizels 1996; Loukas et al. 2000). The major role of TES-120 mucins is the formation of larval surface coat (Page et al. 1992) and protection from antibody and eosinophil attack (Smith et al. 1981; Badley et al. 1987). O-methylated *Toxocara* glycans are specific targets for host antibodies which prove that they are strongly recognised by the host immune system (Fong and Lau 2004; Schabussova et al. 2007; Długosz and Wisniewski 2016). Previously we have shown that mucins stimulate the in vitro production of IL-4, IL-5, IL-6, and TGF-β cytokines by splenocytes from *T. canis* infected mice; however, their effect was less intensive compared with TES antigens (Długosz et al. 2015).

We have also analysed cytokine secretion after cell stimulation with single recombinant mucins (Table 1). In most cases, particular mucins elicited a very similar effect. We only noted two significant differences, one of which was increased TNF-α production after MUC-4 treatment compared with MUC-2 and MUC-3 (Table 1). Apart from other *T. canis* mucins, MUC-4 is either not secreted or is expressed at a relatively low level (Loukas et al. 2000). Moreover MUC-4 shows a serine protease activity and degrades albumin and IgG immunoglobulins (González-Páez et al. 2014). These features may explain the different effect of MUC-4 on TNF-α production by THP-1 cells.

**Table 1:** Cytokine production by THP-1 macrophages stimulated with *T. canis* recombinant glycosylated and deglycosylated mucins

		Glycosylated			Deglycosylated			LPS					
								Glycosylated			Deglycosylated		
TNF-α	MUC-2	286	±	43^ab^	84	±	9^a^	21106	±	855^bef^	26106	±	1623^ah^
	MUC-3	304	±	41^ab^	79	±	6^a^	21609	±	2208^ef^	22700	±	943
	MUC-4	818	±	240^ab^	86	±	22^a^	27179	±	2438^a^	27755	±	2012^ah^
	MUC-5	522	±	245^ab^	69	±	8^a^	26752	±	1739^a^	25602	±	2107^a^
	Control	44	±	10				21420	±	1183			
IL-1β	MUC-2	6568	±	686^ab^	3783	±	491^a^	34556	±	853	35736	±	2438
	MUC-3	6629	±	747^ab^	3688	±	186^a^	35093	±	4850	37014	±	2811
	MUC-4	6755	±	624^ab^	4360	±	686^a^	39517	±	4632	38506	±	4013
	MUC-5	6548	±	892^ab^	4260	±	465^a^	35197	±	2526^b^	42776	±	1857^ah^
	Control	2653	±	212				35389	±	1324			
IL-6	MUC-2	155	±	3^ab^	49	±	37	10740	±	986^a^	10526	±	2023
	MUC-3	189	±	61^a^	84	±	29^a^	10350	±	3472	10334	±	1352^a^
	MUC-4	211	±	19^ab^	40	±	33	11555	±	2723^a^	10107	±	2940
	MUC-5	121	±	49^a^	54	±	15^a^	10995	±	2947	9570	±	3360
	Control	27	±	4				8349	±	866			
IL-10	MUC-2	15	±	3^a^	4	±	3	198	±	18^a^	209	±	8^a^
	MUC-3	16	±	3^a^	3	±	3	190	±	17^a^	202	±	19^a^
	MUC-4	23	±	6^a^	4	±	5	231	±	11^ab^	186	±	8^a^
	MUC-5	22	±	8^a^	8	±	2	232	±	25^ab^	185	±	11^a^
	Control	8	±	3				152	±	8			
IL-12p70	MUC-2	3	±	2	6	±	5	6	±	2^a^	8	±	4^a^
	MUC-3	5	±	4	5	±	3^i^	5	±	1^a^	6	±	0^ai^
	MUC-4	4	±	2	6	±	3^i^	5	±	1^a^	6	±	1^ai^
	MUC-5	4	±	1	10	±	0^a^	4	±	1	17	±	6^a^
	Control	4	±	1				3	±	2			

Results are presented as mean of calculated concentration [pg/ml] ± SD. Statistical analysis was performed by Student’s *t* test. A value of *P* < 0.05 was considered to be significant. Significant differences between groups are marked with: ^a^vs unstimulated cells; ^b^glycosylated vs deglycosylated mucin–stimulated cells; ^c^vs MUC-2-stimulated cells; ^d^vs MUC-3-stimulated cells; ^e^vs MUC-4-stimulated cells; ^f^vs MUC-5-stimulated cells; ^g^vs dMUC-2-stimulated cells; ^h^vs dMUC-3-stimulated cells; ^i^vs dMUC-5-stimulated cells

Another difference was noted in the case of MUC-5, precisely its deglycosylated form which induced significantly higher production of IL-12p70 compared with other mucins (Table 1). MUC-5 protein is probably responsible for the total effect of the mixture of deglycosylated mucins which also upregulated IL-12p70 secretion (Fig. 1). MUC-5 is both larger and more divergent than other mucins, and it is unlikely that it is a member of TES-120 family of surface coat proteins (Doedens et al. 2001). Moreover, MUC-5 ShK/SXC domains are only distantly related to those present in all other *T. canis* mucins. Perhaps the deglycosylation uncovered these unique domains enabling interaction with receptors other than carbohydrate-binding C-type lectin receptors (CLRs) allowing different type of macrophage activation.

Our results clearly show that stimulation of THP-1 cells with mucins induced a glycan-dependent cytokine response. Macrophage treatment with deglycosylated mucins was at least three times less effective comparing to intact glycoprotein molecules (Fig. 1, Table 1). Other studies have already proved that schistosome and filarial glycans are responsible for the induction of Th2 cytokine production (Okano et al. 1999; Tawill et al. 2004). These carbohydrates interact with pattern-recognition receptors (PRRs) on dendritic cells and macrophages, especially C-type lectin receptors (CLRs) which together with toll-like receptors (TLRs) are instrumental in regulation of adaptive immunity (Prasanphanich et al. 2013). Parasitic glycans are bound by several CLRs such as DC-SIGN, mannose receptor (MR), or galactose-type lectin (MGL) (Everts et al. 2012; Meevissen et al. 2012; Klaver et al. 2013). TES antigens were also found to bind human DC-SIGN receptor (Schabussova et al. 2007).

The MGL receptor binds human mucin 1 which becomes overexpressed and aberrantly glycosylated upon malignant transformation. These aberrant glycans on tumour cells may have the ability to suppress antitumor immune responses through activation of the MGL receptor on dendritic cells (Zizzari et al. 2015). It is therefore possible that *Toxocara* mucins also bind MGL and modulate host immune response through such interaction. However, in depth investigations need to be conducted to confirm which CLR receptors might be engaged in immune regulation by *T. canis* molecules.

**Fig. 2:**
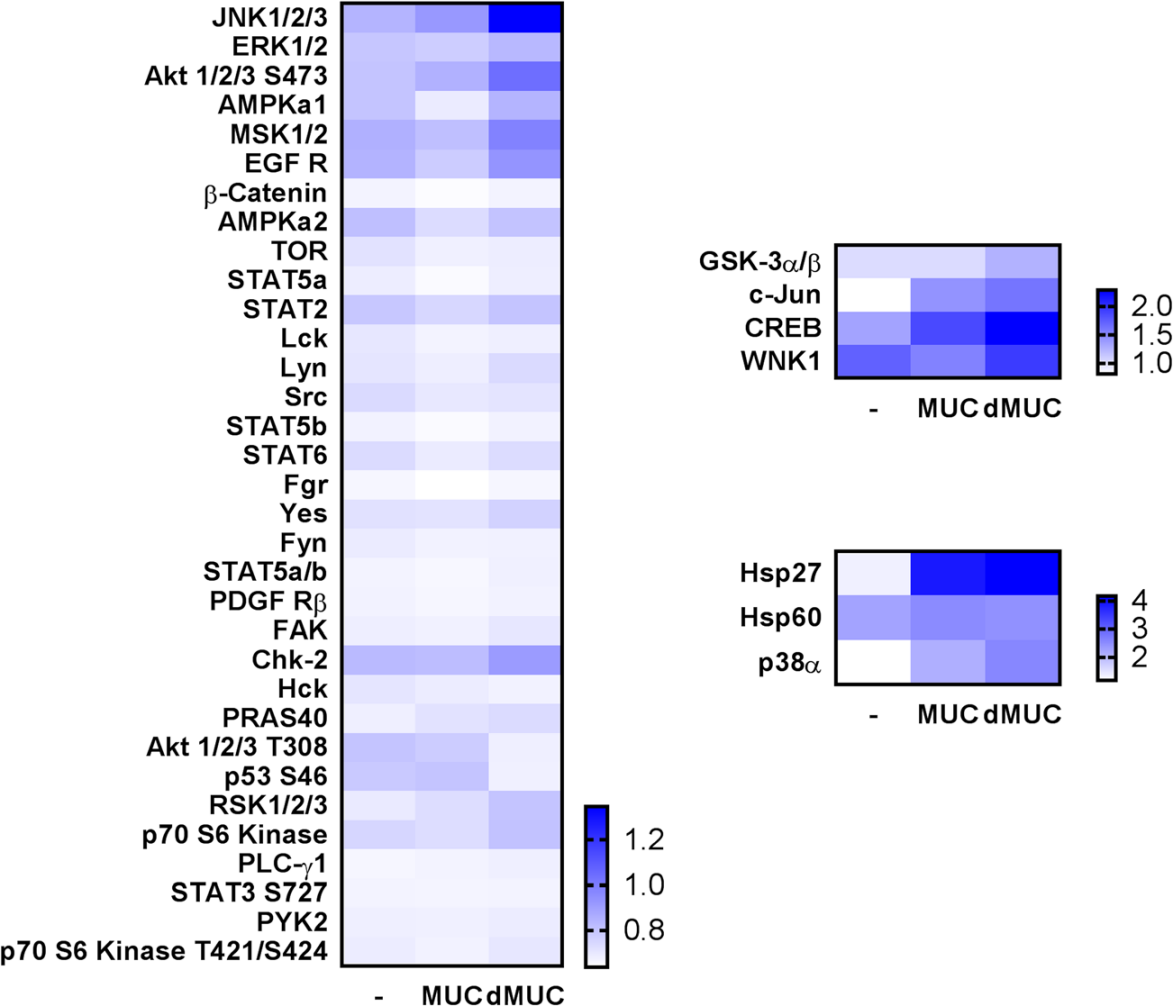
Heatmap of intracellular kinase phosphorylation fold changes measured by the Proteome Profiler Human Phospho-Kinase Array in THP-1 macrophages stimulated with intact and deglycosylated mucins as well as in control unstimulated cells. Results are shown as the adjusted mean volume (OD × mm2) ± SD of two repeats on membrane. The average intensity of the pixels in background volume was calculated and subtracted from each pixel in all standard and unknown

To better understand the influence of glycosylated mucins on cytokine production by THP-1 macrophages, we decided to analyse the phosphorylation profile of kinases involved in signal transduction. The use of the commercial Proteome ProfilerHuman Phospho-Kinase Array enabled a simultaneous analysis of 43 kinase phosphorylation sites. The most intensive, about 4-fold, increase in phosphorylation was observed in the case of heat shock protein 27 (HSP27) in MUC and dMUC-treated cells compared with control (Fig. 2). On contrary, HSP60 was phosphorylated at an equal level in all examined cases. HSPs such as HSP60, HSP70, and HSP90 have been reported to play important roles in antigen presentation, activation of lymphocytes and macrophages, and activation and maturation of dendritic cells (reviewed by Tsan and Gao 2009). HSP27 belongs to small HSPs, and it is intensively upregulated during differentiation from monocytes to macrophages and promotes their survival during inflammation by inhibiting caspase 3 activation, binding to Bcl-2 family members and sequestration of cytochrome c (Voss et al. 2007; Gonzalez-Mejia and Doseff 2009). Moreover, HSP27 induces IL-10 production in human monocytes via activation of p38 signalling independently of TNF-α activation (De et al. 2000). Extracellular HSP27 has been shown to act as a signalling molecule and activate NFkβ in macrophages leading to pro-inflammatory IL-1β and TNF-α as well as anti-inflammatory factors IL-10 and GM-CSF (Salari et al. 2013).

The phosphorylation of MAPK family members such as p38 and JNK as well as c-Jun transcription factor noted in macrophages stimulated by analysed antigens suggest that TLR signalling might be involved (Kawasaki and Kawai 2014). Interestingly, the phosphorylation of these factors was the most intensive in cells stimulated with deglycosylated mucins which suggests that these cells should produce higher amounts of pro-inflammatory cytokines compared with cells treated with intact mucins. However, this was only true for IL-12p70 which was upregulated by deglycosylated mucins (Fig. 1).

This fact might be explained by higher phosphorylation of the CREB transcription factor in these cells. Phosphorylated CREB has been proposed to directly inhibit NF-kβ activation by blocking the binding of CREB binding protein to the NF-kβ complex, thereby limiting pro-inflammatory responses, including the production of IL-2, IL-6, IL-10, and TNF-α (Wen et al. 2010). CREB may be phosphorylated by several kinases i.a. cAMP-dependent protein kinase (AMPK) or pp90 ribosomal S6 kinase (pp90 RSK also known as RSK2) (Wen et al. 2010) both of which were also phosphorylated more intensively in dMUC treated THP-1 cells (Fig. 2). Many reports show that AMPK stimulates SIRT1, PGC-1α, p53, and FoxO factors which can inhibit the NF-kβ signalling with different mechanisms and therefore suppress the process of inflammation and expand the lifespan in diverse types of cells and tissues (reviewed by Salminen et al. 2011).

Our results also show that stimulation of macrophages results in higher phosphorylation of GSK-3 α/β is and less phosphorylation of p53 (Fig. 2). Phosphorylation of GSK-3 α/β results in its inactivation which leads to anti-apoptotic effects and cell survival (Maurer et al. 2014). Phosphorylation of the tumour suppressor p53 at S46 site is more intensive in cells directed toward apoptosis (Smeenk et al. 2011) what again proves that signalling in dMUC-treated cells led to cell survival.

However AMPK activation was not observed in macrophages treated with mucins. In turn, another kinase AKT, also known as protein kinase B (PKB), was activated (Fig. 2). Several points of cross-regulation exist between the PI3K-AKT and AMPK pathways, leading to both reciprocal pathway regulation and convergent regulation of downstream processes (Manning and Toker 2017). Several studies show that AKT kinase pathway is targeted by protozoan parasites leading to the inhibition of apoptosis in infected cells (Chuenkova and PereiraPerrin 2010; Quan et al. 2013; Gupta et al. 2016).

Phosphorylation of two residues (S473 and T308) is required for maximal activation of the AKT kinase (Alessi et al. 1996). These two sites were phosphorylated in MUC-treated cells, whilst in dMUC-stimulated cells only S473 site was phosphorylated (Fig. 2). AKT-mediated phosphorylation of FoxO leads to its binding and cytosolic sequestration by 14-3-3 proteins, thereby attenuating the expression of its gene targets responsible for cell survival, proliferation, and growth (Brunet et al. 2001; Manning and Toker 2017).

PI3K/AKT pathway and its downstream targets have recently emerged as central regulators of activation phenotype in macrophages. It regulates macrophage survival, migration, and proliferation but also orchestrates the response to different metabolic and inflammatory signals in macrophages (Covarrubias et al. 2015). The PI3K/AKT pathway is activated by TLR4 and other pathogen recognition receptors, cytokine and chemokine, and Fc receptors, modulating downstream signals that control cytokine production (reviewed by Vergadi et al. 2017). AKT is crucial for M2 activation as its inhibition abrogates the upregulation of M2 genes (Byles et al. 2013). Several signals such as TGF-β (Gong et al. 2012), IL-10 (Park et al. 2011), and bone morphogenetic protein-7 (BMP-7) (Rocher and Singla 2013) promote M2 polarization via PI3K/AKT signalling.

In conclusion, we have shown that TES molecules, especially mucins induce cytokine production by human THP-1 macrophages. The secretion of cytokines after mucin stimulation was glycan dependent. Sugar moieties also affected the phosphorylation of cellular kinases. Differences were noted especially in the activation of AMPK and AKT kinases in treated cells. The activation of AKT kinase seems crucial for intensive cytokine production.

The difference between cytokine levels secreted after TES and mucin stimulation was very significant. It must be pointed out that although mucins constitute a quite abundant component of TES products, they contain plenty of different molecules which obviously counterbalanced the effect induced by mucins. More efforts should be made to reveal the mechanisms of interaction of particular TES molecules with human immune cells. This will ensure a better understanding of the complex infection process during human toxocariasis.
